# Density-dependency and plant-soil feedback: former plant abundance influences competitive interactions between two grassland plant species through plant-soil feedbacks

**DOI:** 10.1007/s11104-018-3690-x

**Published:** 2018-05-28

**Authors:** Wei Xue, T. Martijn Bezemer, Frank Berendse

**Affiliations:** 1 Department of Terrestrial Ecology, Netherlands Institute of Ecology (NIOO-KNAW), PO Box 50, 6700 AB Wageningen, The Netherlands; 2 Institute of Biology, Section Plant Ecology and Phytochemistry, Leiden University, PO Box 9505, 2300 RA Leiden, The Netherlands; 3 Nature Conservation and Plant Ecology Group, Wageningen University, Droevendaalsesteeg 3a, 6708 PB Wageningen, The Netherlands

**Keywords:** Interspecific competition, Intraspecific competition, Plant abundance, Plant density, Plant-soil feedbacks, Plant-soil interactions, Soil biota

## Abstract

**Backgrounds and aims:**

Negative plant-soil feedbacks (PSFs) are thought to promote species coexistence, but most evidence is derived from theoretical models and data from plant monoculture experiments.

**Methods:**

We grew *Anthoxanthum odoratum* and *Centaurea jacea* in field plots in monocultures and in mixtures with three ratios (3:1, 2:2 and 1:3) for three years. We then tested in a greenhouse experiment the performance of *A. odoratum* and *C. jacea* in pots planted with monocultures and 1:1 mixtures and filled with live and sterile soils collected from the field plots.

**Results:**

In the greenhouse experiment, *C. jacea* produced less aboveground biomass in soil conditioned by *C. jacea* monocultures than in soil conditioned by *A. odoratum* monocultures, while the aboveground biomass of *A. odoratum* in general did not differ between the two monospecific soils. The negative PSF effect was greater in the 1:1 plant mixture than in plant monocultures for *A. odoratum* but did not differ for *C. jacea*. In the greenhouse experiment, the performance of *C. jacea* relative to *A. odoratum* in the 1:1 plant mixture was negatively correlated to the abundance of *C. jacea* in the field plot where the soil was collected from. This relationship was significant both in live and sterile soils. However, there was no relationship between the performance of *A. odoratum* relative to *C. jacea* in the 1:1 plant mixture in the greenhouse experiment and the abundance of *A. odoratum* in the field plots.

**Conclusions:**

The response of a plant to PSF depends on whether the focal species grows in monocultures or in mixtures and on the identity of the species. Interspecific competition can exacerbate the negative plant-soil feedbacks compared to intraspecific competition when a plant competes with a stronger interspecific competitor. Moreover, the abundance of a species in mixed plant communities, via plant-soil feedback, negatively influences the relative competitiveness of that species when it grows later in interspecific competition, but this effect varies between species. This phenomenon may contribute to the coexistence of competing plants under natural conditions through preventing the dominance of a particular plant species.

**Electronic supplementary material:**

The online version of this article (10.1007/s11104-018-3690-x) contains supplementary material, which is available to authorized users.

## Introduction

Plants can alter soil abiotic and biotic properties. These changes in the soil can subsequently influence the performance of the same or other plant species that grow on this substrate, which is known as plant-soil feedback (Bever et al. 1997; Ehrenfeld et al. 2005; van der Putten et al. 2013). Plant-soil feedbacks can influence plant growth positively through the accumulation of soil nutrients (Berendse 1990; Chapman et al. 2006; Wardle et al. 1999) or symbiotic mutualists (Klironomos 2002; van der Putten et al. 2016) and negatively by nutrient immobilization or depletion (Berendse 1994), or accumulation of soil pathogens (van der Putten et al. 2016). Positive feedbacks are thought to promote plant dominance and homogenize plant communities (Hartnett and Wilson 1999; O’Connor et al. 2002), while negative feedbacks allow species coexistence and increase plant species diversity (Bever 2003; Bever et al. 1997; Petermann et al. 2008).

Plant-soil feedbacks of a particular plant species are generally tested by comparing the performance of that species on soils that were planted with monocultures of the same species and on soils planted with monocultures of the other species (Bever et al. 1997; Brinkman et al. 2010; Kulmatiski et al. 2008). However, in the field, plants rarely grow in monocultures and often compete with other plant species. The presence of other species will not only affect the soil resources that are available for each of the component species (Casper and Jackson 2002; Hawkes et al. 2005), but also affect the composition of the soil community including both mutualistic and pathogenic organisms (Bartelt-Ryser et al. 2005; Eisenhauer 2016; Hausmann and Hawkes 2009). Conspecific plant-soil feedback effects may be weaker in soils conditioned by plant mixtures than in soils conditioned by monocultures of that species, due to the lower abundance of the focal species in plant mixtures (Hawkes et al. 2013). Several studies have suggested that negative plant-soil feedbacks in natural systems might be density-dependent (Bagchi et al. 2010; Bell et al. 2006; Comita et al. 2014; Kos et al. 2013; van de Voorde et al. 2011). If the local density of a species in a plant community increases, species-specific soil pathogens are expected to increase as well, thus decreasing the per capita fitness of that plant species (Bagchi et al. 2010). However, only one study has empirically examined this so far (Dudenhöffer et al. 2018).

The response of a plant to plant-soil feedback depends on whether the plant grows individually or in competition with other plants, and several studies have shown that negative plant-soil feedbacks are generally enhanced in the presence of competitors (e.g. Callaway et al. 2004; Hol et al. 2013; Kulmatiski et al. 2008; Shannon et al. 2012). However, whether plants grown in monocultures (and hence experience intraspecific competition) and grown in plant mixtures (and experience interspecific competition) respond differently to plant-soil feedbacks is poorly understood. Casper and Castelli (2007) proposed that negative conspecific plant-soil feedbacks are expected to be more pronounced when plants compete with the same species (intraspecific competition) than with other plant species (interspecific competition). However, Kardol et al. (2007) and Petermann et al. (2008) reported that the negative response of plants to plant-soil feedback is stronger when the plants grew in interspecific competition than when grew in intraspecific competition. Recently, Jing et al. (2015) demonstrated that when two plants compete, the soil feedback effects of one species can negatively influence the other competing species more than that it influences the conspecific species, even though the conspecific species suffers from negative plant-soil feedbacks when grown alone. Hence, in interspecific competition, it can be either advantageous or disadvantageous for a species to grow in the soil conditioned by the same species (Jing et al. 2015). It remains unresolved how the density of a plant species in a mixed plant community, via plant-soil feedback, influences the competition between two plant species when they grow later in the soil where these plants were previously growing.

Many plant-soil feedback studies compare the growth of a plant in sterilised soils with and without addition of a soil inoculum (e.g. van der Putten et al. 1993; Kardol et al. 2006; Kos et al. 2013). However, adding a small amount of live soil to a large amount of sterilised bulk soil may not result in representative soil communities. For example, the density of the soil community may be very different from that observed outdoors in the field (Brinkman et al. 2010). Alternatively, plant-soil feedbacks can be determined by comparing plant growth in sterilised and unsterilised pure soils. Sterilising typically results in increased availability of soil nutrients (Brinkman et al. 2010). Plant-soil feedbacks can be driven simultaneously by abiotic and biotic changes in the soil. Therefore, the difference between the performance of a plant in unsterilised and sterilised soil would be a net effect of the elimination of soil biota and the increase in soil nutrients.

The aim of the present study was to investigate how, via plant-soil feedbacks, the abundance of a species in a plant community consisting of two species influences the growth and competition between these two species when they grow later in the soil. We grew the grass *Anthoxanthum odoratum* and the forb *Centaurea jacea* in field plots in monocultures and in mixtures with three ratios (3:1, 2:2 and 1:3) for three years. We then tested the performance of *A. odoratum* and *C. jaceae* in a greenhouse experiment with the two species grown in monocultures and in 1:1 mixtures in pots filled with either unsterilised or sterilized soils collected from the field plots. We specifically hypothesised that: (1) plants will produce less biomass in “own” soil (conditioned by conspecific monocultures) than in “foreign” soil (conditioned by heterospecific monocultures) as conspecific plant-soil feedbacks are generally negative. As a consequence, when two plant species grow in mixtures (interspecific competition), the plant species that encounters its “own” soil will be at competitive disadvantage. (2) Negative conspecific plant-soil feedback effects will increase with a greater abundance of that species during the previous growth phase (i.e. conditioning phase) in the plant community, e.g. due to the build-up of species-specific soil pathogens in the soil. (3) Negative conspecific plant-soil feedbacks will be stronger when plants grow in 1:1 plant mixtures than when they grow in monocultures, as the competing species will not suffer from negative plant-soil feedbacks and hence will be a stronger competitor. (4) Plant-soil feedbacks will be stronger in live soil than in sterile soil as sterilization will eliminate the soil biota that can drive the plant-soil feedbacks.

## Methods and materials

### Plant species

Our study species were *Anthoxanthum odoratum* L. (Poaceae) and *Centaurea jacea* L. (Asteraceae). *A. odoratum* is a perennial grass that produces closely connected ramets, while *C. jacea* is a long-lived perennial herb that has monocarpic shoots and can form extensive belowground branches (Jongejans and de Kroon 2005). Both species are native grassland species in western Europe. They have similar life history strategies, i.e., with clonal growth as well as sexual reproduction (Hartemink et al. 2004) and commonly coexist in meadows (van Ruijven and Berendse 2003).

### Garden experiment

We performed a long-term competition experiment with *A. odoratum* and *C. jacea* in field plots from April 2013 to September 2015. In this experiment, we planted *A. odoratum* and *C. jacea* in monocultures as well as in mixtures at three planting ratios (3:1, 2:2 and 1:3) in plots (1 × 1 m^2^). Black soil (total N: 2.13 g kg^−1^; total C: 28.2 g kg^−1^; total P: 2.39 g kg^−1^) was used in each plot. Soils collected from these experimental plots were coded as Ao soil (conditioned by monocultures of *A. odoratum*), Cj soil (conditioned by monocultures of *C. jacea*), 3Ao/1Cj soil (conditioned by a 3:1 mixture of *A. odoratum* and *C. jacea*), 2Ao/2Cj soil (conditioned by a 2:2 mixture of *A. odoratum* and *C. jacea*) and 1Ao/3Cj soil (conditioned by a 1:3 mixture of *A. odoratum* and *C. jacea*), respectively. The total number of seedlings planted in each plot was 144. Each treatment had five replicate blocks, yielding 25 plots. Plots were weeded regularly. In September 2015, we clipped each plant in the central 60 × 60 cm^2^ at a height of 1 cm and determined the aboveground biomass of each of the two plant species in each plot after oven-drying it to constant weight. In February 2016, we collected the soil from the central area of 60 × 60 cm^2^, to a depth of 20 cm of each experimental plot. The soil from each plot was sieved (1.5 cm mesh) and separated in two parts. Half of the soil from each plot was sterilized by γ-irradiation (minimum 25KGray, Isotron, Ede, the Netherlands) so that there were 50 different conditioned soils (5 planting treatments × 2 sterilization treatments × 5 replicate blocks).

### Greenhouse experiment

**Fig. 1:**
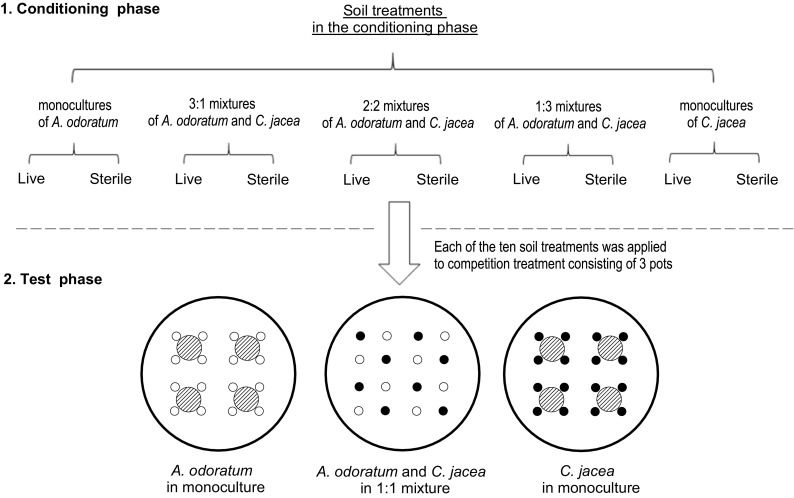
Schematic representation of the experimental design. (1) Conditioning phase: conditioned soils were collected from a three-year field experiment, in which soils were conditioned separately by monocultures of *A. odoratum* and *C. jacea*, as well as mixtures of these two species at three planting ratios (3:1, 2:2 and 1:3). Conditioned soils were either sterilized or not (i.e., live and sterile), resulting in 10 soil treatments. (2) Test phase: we planted either 16 plants of species *A. odoratum* or species *C. jacea* in monocultures, or eight plants of each of the species in mixtures in each of the ten soil treatments in a greenhouse experiment. White and black dots represent the initial positions where *A. odoratum* and *C. jacea* were grown. The shaded circles within each pot represent the positions where we took soil samples

Each of the 50 soil samples was used to fill three pots (21 cm in top-diameter and 18 cm in height) with 5.6 kg soil in each pot (Fig. 1) so that the entire experiment consisted of 150 pots. Pots filled with soils collected in the same field block were allocated to the same block in the greenhouse experiment so that there were five blocks corresponding to the blocks in the field experiment. Pots of different treatments were randomized within each block. Before filling the pots, we placed a piece of filter paper (15 cm in diameter) at the bottom of each pot to prevent soil from passing through holes in the bottom of the pot but allowing vertical movement of water. Each pot was placed on a tray to prevent possible contamination through leachate. For soil chemical analysis, we randomly selected three blocks, and took soil samples (5 planting treatments × 2 sterilization treatments) from each selected block. Unsterilised soils (not sterilised by γ-irradiation; live soil) and sterile soils (sterilized by γ-irradiation) were analysed separately. We measured soil organic matter content, nutrient content (NH_4_, NO_3_ and PO_4_), water content and pH (Table 1; Methods S1).

**Table 1:** Abiotic characteristics of live and sterile soils collected from the field plots

Sterilization	Soil	P-PO_4_ (mg/kg)	N-NO_3_ (mg/kg)^1^	N-NH_4_ (mg/kg)	pH (H_2_O)	Moisture (%)	Organic Matter (%)
Live soils	Ao soil	0.00 ± 0.00^d^	0.49	6.14 ± 4.00^b^	6.85 ± 0.11^b^	14.45 ± 1.64	2.94 ± 0.48
	3Ao/1Cj soil	0.16 ± 0.12^bcd^	0.50 ± 0.14	6.74 ± 4.91^b^	6.97 ± 0.16^ab^	12.41 ± 0.58	2.41 ± 0.18
	2Ao/2Cj soil	0.06 ± 0.06^cd^	0.57 ± 0.14	5.23 ± 3.80^b^	6.95 ± 0.15^ab^	15.03 ± 1.13	2.77 ± 0.29
	1Ao/3Cj soil	0.06 ± 0.06^cd^	0.56 ± 0.27	7.02 ± 4.99^ab^	7.02 ± 0.11^ab^	11.98 ± 2.04	2.50 ± 0.58
	Cj soil	0.25 ± 0.12^bcd^	0.66 ± 0.25	5.76 ± 3.89^b^	6.98 ± 0.20^ab^	13.71 ± 0.77	2.63 ± 0.16
Sterile soils	Ao soil	0.67 ± 0.21^abc^	0.90 ± 0.33	23.78 ± 7.76^a^	7.06 ± 0.05^ab^	13.13 ± 2.26	2.77 ± 0.77
	3Ao/1Cj soil	0.22 ± 0.12^bcd^	0.50 ± 0.08	13.65 ± 1.30^ab^	7.15 ± 0.10^a^	11.53 ± 0.72	2.15 ± 0.17
	2Ao/2Cj soil	0.73 ± 0.15^ab^	0.54 ± 0.25	20.60 ± 1.13^ab^	7.19 ± 0.08^a^	11.34 ± 0.88	2.04 ± 0.14
	1Ao/3Cj soil	0.49 ± 0.16^abcd^	0.58 ± 0.30	17.54 ± 0.82^ab^	7.16 ± 0.07^a^	10.28 ± 1.26	1.77 ± 0.23
	Cj soil	1.09 ± 0.23^a^	0.47 ± 0.08	20.68 ± 5.17^ab^	7.17 ± 0.11^a^	14.75 ± 2.06	2.60 ± 0.55
ANOVA	Sterilization	37.10***	0.47	29.71***	21.17***	2.42	3.08
	Soil	3.36*	1.46	0.41	1.40	1.88	1.33
	Interaction	2.36	0.26	0.64	0.13	0.82	0.45

Means (±SE), and *F*- and *P*-values of two-way ANOVAs are given. Mean values sharing the same superscript (a-d) are not significantly different among the ten soils in each column (Tukey post hoc tests). Ao soil and Cj soil represent soils conditioned by monocultures of *A. odoratum* and *C. jacea*, respectively, while 3Ao/1Cj soil, 2Ao/2Cj soil and 1Ao/3Cj soil represent the soils conditioned by 3:1, 2:2 and 1:3 mixtures of *A. odoratum* and *C. jacea*, respectively. ^***^ *P* < 0.001, ^**^ *P* < 0.01, ^*^ *P* < 0.05

^1^Data was log-transformed. Data of N-NO_3_ in the live soil conditioned by monoculture of *A. odoratum* (Ao soil) was based on only one sample

We purchased seeds of *A. odoratum* and *C. jacea* from a specialized company (Cruydthoeck, Nijeberkoop, the Netherlands). All seeds of each plant species were evenly sown on plastic trays filled with steamed potting soil (0.03 N-0.03P-0.03 K, Seed Starting Potting Mix, Miracle-Gro Lawn Products, Inc., Marysville) that facilitates fast root development in a heated greenhouse (20.0 °C average temperature, 70.2% average relative humidity). The trays were watered daily. One week after germination, the trays with seedlings were moved to an unheated greenhouse (12.8 °C average temperature, 70.3% average relative humidity) until they were transplanted into the pots.

We planted similar-sized seedlings of *A. odoratum* and *C. jacea* in each pot in either monocultures or 1:1 mixtures (Fig. 1). In monocultures, we planted 16 seedlings of *A. odoratum* or *C. jacea* in each pot. In the 1:1 mixtures, we planted eight seedlings of *A. odoratum* and eight seedlings of *C. jacea* in alternating positions (Fig. 1). After one week, we replaced dead seedlings. All other species emerging from the seed bank of the soil were removed manually during the experiment.

The experiment was maintained for 90 days (from 11 April to 11 July 2016) in the same unheated greenhouse. During the experiment, the mean temperature and the relative humidity in the greenhouse were 17.4 °C and 67.5%. Water was added to all pots three times per week.

### Harvest and measurement

Ninety days after transplanting, we clipped all plants at the soil level. The two different plant species in the 1:1 mixtures were harvested separately. We also took four soil cores (4.0 cm diameter, straight down to the bottom of pot) in each pot to measure the root mass (Fig. 1). We only took soil cores from pots planted with monocultures because it was not possible to separate roots of the two different plant species in the mixtures. We then washed all root samples over a 0.5 mm sieve. Aboveground parts and belowground parts of each plant species in each pot were oven-dried at 70 °C for 48 h and weighted.

### Data analysis

In the garden experiment, we used replacement diagrams to show the biomass of the two species in all planting treatments (i.e., monocultures and mixtures at three different ratios: 3:1, 2:2 and 1:3), and used the relative crowding coefficient (*k*) of a species to assess its competitive ability in the mixtures relative to that in the monocultures (De Wit 1960). We calculated *k* as: $\left(\left(z-1\right)/z\right)\left(O/M\right){\left(\frac{O}{M}-1\right)}^{-1}$, in which *z* is the planting frequency of a species in the mixtures, *O* and *M* are the aboveground biomass of that species in mixtures and in monocultures, respectively. We then used linear regressions to assess whether *k* was dependent on the planting frequencies of each species.

In the greenhouse experiment, we first calculated aboveground biomass per plant of *A. odoratum* and *C. jacea* in each pot, since monocultures had twice as many individuals per species at the start of the experiment as mixtures. As belowground biomass was determined by taking soil cores, it was calculated as the mean of the root biomass in the four soil cores and not as the root biomass per pot. Data of aboveground and belowground biomass were log transformed to improve the normality and homogeneity of variance.

We first analysed the aboveground and belowground biomass of each plant species on the two soils collected from the monospecific plots in the field (i.e., Ao soil and Cj soil collected from the garden experiment; monospecific soil) to test whether there was a soil type effect on the performance of the two species. We first performed a full-model analysis including species (*A. odoratum* vs. *C. jacea*), soil type (soil type was tested as “own” soil vs. “foreign” soil), sterilization (live vs. sterile), competition (monocultures vs. mixtures; only for aboveground biomass) and their interactions as fixed factors, with block as random factor. Subsequently, we separately analysed biomass of each species using a mixed-effect ANOVA with soil type, sterilization, competition (only for aboveground biomass) and their interactions as fixed factors, and block as a random factor.

To test if plant-soil feedback (PSF) differed in response to competition mode and sterilization treatment, we calculated the PSF as the log-ratio of plant biomass on “own” and “foreign” soils ($\ln \frac{Biomass_{own}}{Biomass_{foreign}}$) for each combination of species, competition and sterilization. PSFs were calculated separately for each replicate and based on aboveground biomass (aboveground PSF) and belowground biomass (belowground PSF). We analysed the PSF-values using a full-model analysis including species, sterilization, competition (only for aboveground PSF) and their interactions as fixed factors, with block as random factor, followed by separate analyses using mixed-effect ANOVA with sterilization, competition (only for aboveground PSF) and the interaction (only for aboveground PSF) as fixed factors, and block as a random factor.

Since in the pots planted with plant mixtures the growth of the two species is not independent, we evaluated the competition between the two species by calculating for each pot the competitive balance index (CB). The CB was calculated as: $\ln \frac{MIX_{Ao}}{MIX_{Cj}}$ with *MIX*_*Ao*_ and *MIX*_*Cj*_ representing the biomass of *A. odoratum* and *C. jacea* in the 1:1 mixtures in the greenhouse experiment, respectively. Using this index, the performance of the two species in a pot was combined. CB will be equal to zero if the two species perform equally well in mixtures; CB will be positive if the biomass of *A. odoratum* is higher than *C. jacea* and negative if *C. jacea* biomass is higher. A one-sample *t*-test was used to test whether the CB differed from zero. We used two-way ANOVA to test the effects of soil type (Ao soil vs. Cj soil), sterilization and their interaction on the CB on the two monospecific soil types. Block was included as a random factor. Means of the CB values on different monospecific soils were compared using a post-hoc test for pairwise comparison.

Further, the relationship between the growth of either *A. odoratum* or *C. jacea* in the greenhouse experiment and its former abundance in the field plots was analysed using linear regression for each species, sterilization and competition (only for aboveground biomass) combination. The relationship between the CBs between the two species in the greenhouse experiment and the former abundance of either *A. odoratum* or *C. jacea* in the field plots was also analysed using linear regression separately for live and sterile soils.

We performed all data analysis using R (version 3.3.2) (http://www.r-project.org) in RStudio (version 1.0.44) (http://rstudio.org). Linear mixed-effect models were fitted with *nlme* (version 3.1–128) (Pinheiro et al. 2016). Post-hoc comparisons were tested as planned contrasts using the *multcomp* package (version 1.4–6) in R (Hothorn et al. 2008). All data were checked graphically for normality and homogeneity for variance.

## Results

### Biomass of the two plant species and soil properties in the field plots

In all mixtures, the total aboveground biomass of *C. jacea* per plot was significantly greater than that of *A. odoratum* (Fig. S1). *A. odoratum* showed an inverse sigmoid curve in the replacement diagram (Fig. S1) and its competitive ability (relative crowding coefficient, *k*) decreased with increasing frequency (Fig. S2A). There was no significant relationship between the competitive ability of *C. jacea* and its planting frequency (Fig. S2B).

The amount of P-PO_4_, N-NH_4_, and the pH (H_2_O) were significantly higher in sterile soil than in live soil but there was no difference in the amount of N-NO_3_, in soil moisture and in organic matter between live and sterile soils (Table 1). Overall, none of the measured properties except P-PO_4_ was different among the five soils (Ao soil, 3Ao/1Cj soil, 2Ao/2Cj soil, 1Ao/3Cj soil and Cj soil; Table 1). The amount of P-PO_4_ was overall higher in Cj soil than in other soils (Table 1).

### Plant-soil feedback effects in monospecific soils in the greenhouse experiment

**Fig. 2:**
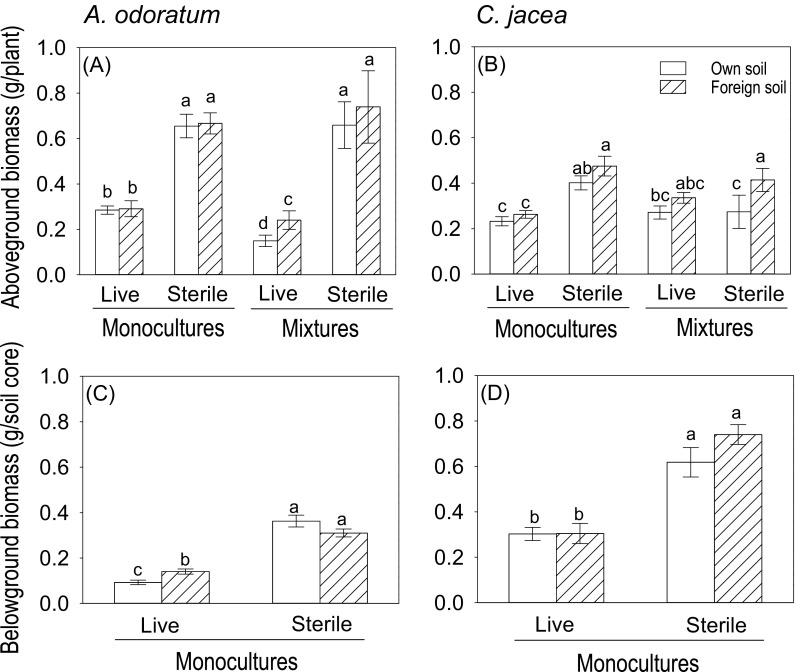
Aboveground biomass per plant (**a** and **b**), and belowground biomass per soil core (**c** and **d**) of *A. odoratum* (**a** and **c**) and *C. jacea* (**b** and **d**) on “own” (soil conditioned by monocultures of the same species) and “foreign” soils (soil conditioned by monocultures of the other species) in the greenhouse experiment. “Sterile” and “Live” indicate sterilized soil and non-sterilized soil respectively. Plants were grown in monocultures and in 1:1 mixtures in the greenhouse experiment. Mean values (± 1 SE) are presented. Letters above the bars indicate significant differences in aboveground biomass among each panel

In the greenhouse experiment, *C. jacea* overall produced less aboveground biomass when grown in “own” soil (Cj soil) than in “foreign” soil (Ao soil), while aboveground biomass of *A. odoratum* did not differ between the two soils (Table S1A, S2A; Fig. 2a, b). *A. odoratum* produced more aboveground biomass in sterile soil than in live soil in both plant monocultures and the 1:1 plant mixture, but the difference was much bigger in the 1:1 plant mixture than in plant monocultures (Table S2A; Fig. 2a). In monocultures, *C. jacea* also produced more aboveground biomass in sterile soil than in live soil, but in the 1:1 plant mixture, there was no difference between these two sterilization treatments (Table S2A; Fig. 2b).

The belowground biomass of *A. odoratum* was significantly greater in live “foreign” soil than in live “own” soil, but did not differ in sterile “foreign” and “own” soil (Table S2B; Fig. 2c). In contrast, belowground biomass of *C. jacea* did not differ between “foreign” and “own” soil in either live or sterile soils (Table S1B, S2B; Fig. 2d).

The aboveground PSF of *A. odoratum* tended to be lower in the1:1 plant mixture than in the plant monoculture independent of sterilization treatment (Table S3A; Fig. S3A). Generally, the aboveground PSF of *C. jacea* was negative, but there was no difference between plant monocultures and 1:1 plant mixtures (Table S3A; Fig. S3B). The belowground PSF of *A. odoratum* was significantly greater in sterile soil than in live soil but there was no difference between these two soil types for *C. jacea* (Table S3B; Fig. S3C, D).

### Competitive balance between the two species grown in the 1:1 plant mixture in monospecific soils in the greenhouse experiment

**Fig. 3:**
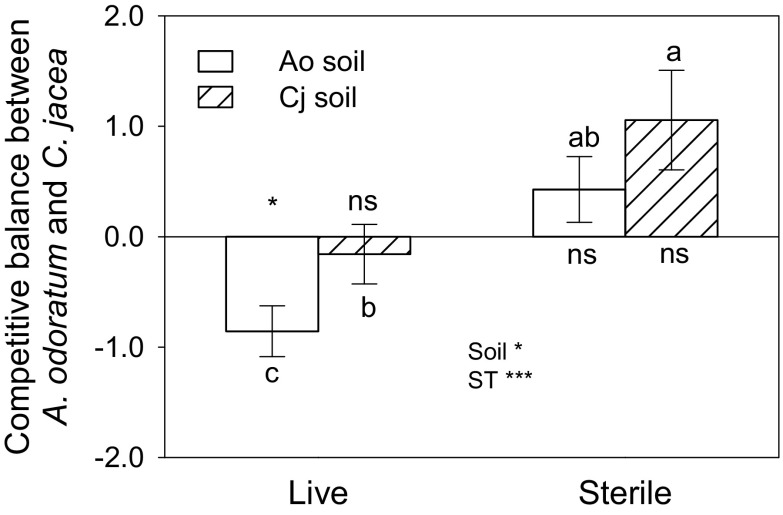
Competitive balance (CB; $\ln \frac{MIX_{Ao}}{MIX_{Cj}}$) between *A. odoratum* and *C. jacea* in the 1:1 mixture on Ao soil (soils collected from field plots with *A. odoratum* monocultures) and Cj soil (soils collected from field plots with *C. jacea* monocultures) in the greenhouse experiment in sterile and live soil. Mean values (± 1 SE) and significant effects of a two-way ANOVA with soil type (Soil), sterilization (ST) and the interaction are presented, the superscript asterisks give *P*: * *P* < 0.05 and *** *P* < 0.001. Bars that share the same letters are not significant different based on a Tukey post-hoc comparison. Negative CB values indicate that the biomass of *C. jacea* is higher than that of *A. odoratum*, while positive CB values indicate that *A. odoratum* biomass is higher. The asterisk at the start of the first bar indicate that the values differ from zero (*P* < 0.05) based on a one-sample *t*-test, ns indicates not significant

The competitive balance index (CB: performance of *A. odoratum* in the 1:1 mixture relative to that of *C. jacea*) was greater in sterile soil than in live soil (Fig. 3). Overall, *C. jacea* was superior to *A. odoratum* in live soil while the reverse was true in sterile soil (Fig. 3). The competitive balance, CB was significantly smaller in live Ao soil than in live Cj soil, but there was no significant difference in CB between sterile Ao and sterile Cj soil. CB was significantly smaller than zero in live Ao soil, but did not differ from zero in live Cj soil, indicating that *C. jacea* was competitively superior in live Ao soil, but not in live Cj soil. In sterile soil, the pattern was similar but *A. odoratum* was superior over *C. jacea* (Fig. 3).

### Relationships between the growth and competitive balance in the greenhouse and abundance (biomass) in the field plots

For both species, there was no significant relationship between the growth (aboveground biomass and belowground biomass) of the species in the greenhouse experiment and the abundance of either species in the field experiment (all *P* > 0.05; Fig. S4-S6).

**Fig. 4:**
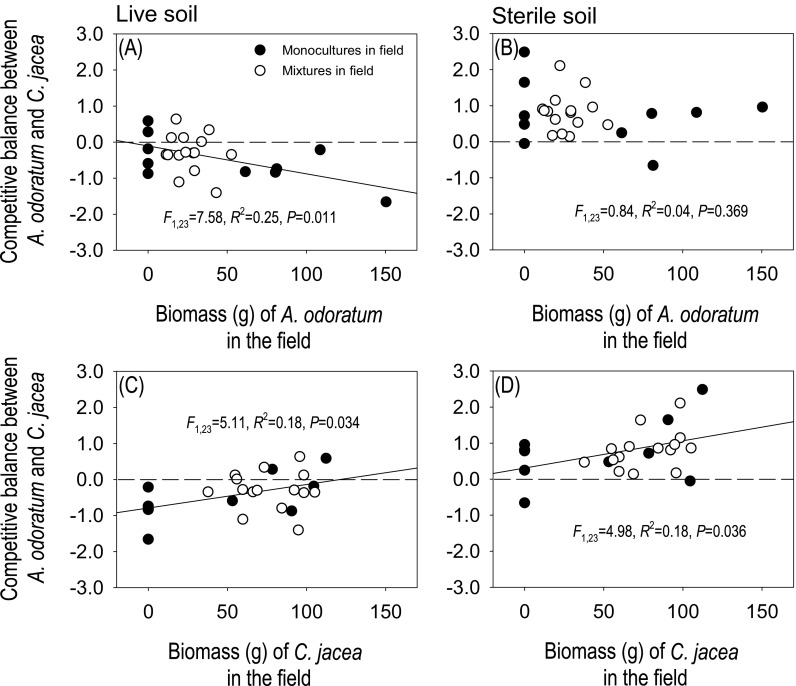
Relationship between the biomass of *A. odoratum* (**a** and **b**) or *C. jacea* (**c** and **d**) in the field plots and the competitive balance (CB;$\ln \frac{MIX_{Ao}}{MIX_{Cj}}$) between *A. odoratum* and *C. jacea* in the 1:1 plant mixture in the greenhouse experiment. For the CB, negative values indicate that the biomass of *C. jacea* is higher than that of *A. odoratum*, while positive values indicate *A. odoratum* biomass is higher. Black and white dots represent soils collected from field plots planted with monocultures and mixtures, respectively. The *F*-, *R*^2^- and *P*-values obtained from linear regressions are also presented

The CB between *A. odoratum* and *C. jacea* in the greenhouse experiment was negatively correlated to the abundance of *A. odoratum* in the field experiment in live soil (Fig. 4a), but not in sterile soil (Fig. 4b). However, this pattern was caused by one data point and was no longer significant after removing this point (Fig. S7). There was a significant positive relationship between the CB between *A. odoratum* and *C. jacea* in the greenhouse experiment and the abundance of *C. jacea* in the field experiment in both live (Fig. 4c) and sterile soil (Fig. 4d).

## Discussion

In the present study, we show that the competitive balance (the performance of *A. odoratum* relative to *C. jacea*) in the greenhouse was related to the former abundance of *C. jacea*, while it was independent of the former abundance of *A. odoratum* in the field. This result implies that the abundance of a plant species in mixed communities can influence the competitive interactions of later growing plants via plant-soil feedback effects, but that these effects vary between species.

Negative plant-soil feedback strength is often assumed to increase with previous plant density, but this has been rarely tested (e.g., Bagchi et al. 2010; Bell et al. 2006; Comita et al. 2014; Kos et al. 2013). In the present study, we therefore expected a negative relationship between the growth of a species in the greenhouse experiment and its former abundance in the field. However, we did not find such a relationship for either of the two species. A possible explanation is that *C. jacea* was competitively superior in all field plots and produced much more biomass than *A. odoratum*, even at low planting densities (Fig. S1). Hence, *C. jacea* may have played a dominant role in conditioning the soils in all mixtures, which may explain the lack of a relationship between the growth of a species in the greenhouse experiment and its former abundance in the field. However, it is also important to note that a low-productive plant species may have a much larger impact on the soil than a highly productive species. Another possible explanation may relate to the non-additivity of plant-soil feedbacks (Hawkes et al. 2013; Kuebbing et al. 2014). Growth of a test plant species in soil conditioned by different plant species simultaneously is not necessarily the same as the averaged effects those species have in monocultures.

We expected that the former plant abundance of one species may have a negative influence on its competitive performance later on. Indeed we observed that there was a negative relationship between the density in the field and the competitive performance for both *A. odoratum* and *C. jacea* although the significance of the relationship for *A. odoratum* was determined by one data point*.* In the present study, the two plants may have conditioned the soil differently, it is possible that the conditioning effects of *A. odoratum* on the soil were overall weak even though its biomass varied strongly among the field plots (Fig. S4).

Remarkably, the negative effects of the former abundance of *C. jacea* on its competitive performance occurred both in live and sterile soils. In this study, *A. odoratum* benefited more from higher soil nutrient availability than *C. jacea* as indicated by the positive impact of sterilization on the performance of *A. odoratum* relative to *C. jacea*. Potentially, *C. jacea* which was more productive than *A. odoratum* in the field may have produced more labile soil organic matter leading to increased soil nutrient availability (Berendse 1990), which, in turn, could favour *A. odoratum* more than *C. jacea* (Fig. S8). However, in our study, we did not observe an increase in availability of nutrients in *C. jacea* soils (Table 1). Alternatively, we speculate that these negative plant-soil feedback effects may be driven by allelopathic effects (Callaway and Aschehoug 2000). It is possible that the root exudates of *C. jacea* in both live and sterile soils reduced the performance of *C. jacea* relative to *A. odoratum.* Our results would then suggest that allelopathy may inhibit or slow down the dominance of a particular species in interspecific competition, promoting the coexistence of species.

We hypothesized that negative plant-soil feedbacks would be stronger in the 1:1 plant mixture than in plant monocultures (Kardol et al. 2007; Petermann et al. 2008; van der Putten and Peters 1997). We found only limited evidence for this. The performance of *A. odoratum* was reduced more in live “own” soil than in live “foreign” soil when grown in competition i.e., in the 1:1 plant mixture than when grown in plant monoculture and a similar trend was observed for *C. jacea*. In the 1:1 plant mixture in the greenhouse experiment, *C. jacea* was competitively superior to *A. odoratum* in live soil, and the performance of *C. jacea* in its “own” soil was less reduced when grown together with *A. odoratum*, while the performance of *A. odoratum* in its “own” soil was much more reduced when grown with *C. jacea*. These results would suggest that interspecific competition can exacerbate negative plant-soil feedback effects compared to intraspecific competition, but only when a plant competes with a stronger competitor.

We expected that negative plant-soil feedbacks would be stronger in live soil than in sterile soil. In agreement with our hypothesis, the negative plant-soil feedback effects of *A. odoratum* were smaller or less negative in sterile soil than in live soil, but for *C. jacea* this was not true. This result indicates that the negative feedbacks encountered by *A. odoratum* appears to be biotic while that by *C. jacea* is abiotic. However, it should be noted that sterilization of soils can change soil features such as nutrient availability (Brinkman et al. 2010; Jakobsen and Andersen 1982; Powlson and Jenkinson 1976), and fast-growing species of microorganisms can develop rapidly in sterilized soil (Brinkman et al. 2010; de Boer et al. 2003). Overall, our results regarding the effects of sterilization on plant-soil feedbacks effects are inconclusive, even though, sterilization per se, had a large effect on plant growth and plant competition.

We conclude that conspecific plant-soil feedbacks can negatively influence plant growth and that the negative effects tend to be stronger when the test plants grow in interspecific competition than when they grow in intraspecific competition. Moreover, the former abundance of a species in mixed plant communities, via plant-soil feedback, can negatively influence the relative competitiveness of that species when it grows later in interspecific competition. However, our study also shows that these plant-soil feedback effects depend on the identity of the plant species. In a broader context, the density dependent feedback effects may prevent the dominance of one species and promote the coexistence of competing plant species in natural systems.

## Electronic supplementary material

ESM 1 (DOCX 90 kb)
