# Supplementary material for: Density-dependency and plant-soil feedback: former plant abundance influences competitive interactions between two grassland plant species through plant-soil feedbacks
Source: Plant Soil. 2018 May 28;428(1):441–52. doi: 10.1007/s11104-018-3690-x (PMC6435205; doi:10.1007/s11104-018-3690-x)
Supplement: Supplementary file 1 — (DOCX 90 kb) [file 11104_2018_3690_MOESM1_ESM.docx]

**Supporting information**

**Table S1.** Full model results of mixed-effect ANOVA testing the effects of species (*A. odoratum* vs. *C. jacea*), soil type (“own” soil vs. “foreign” soil), sterilization (live vs. sterile) and competition (monocultures vs. mixtures; only for aboveground biomass) on aboveground biomass (A) and belowground biomass (B) of *A. odoratum* and *C. jacea* in the greenhouse experiment. DF, DenDF, *F*-, and *P*- values are given.

|  | DF | DenDF | *F* | *P* |
| --- | --- | --- | --- | --- |
| (A) *Aboveground biomass*^1^ | | |  |  |
| Species (SP) | 1 | 60 | 9.23 | **0.004** |
| Soil type (Soil) | 1 | 60 | 9.74 | **0.003** |
| Sterilization (ST) | 1 | 60 | 108.02 | **<0.001** |
| Competition (C) | 1 | 60 | 5.22 | **0.026** |
| SP × Soil | 1 | 60 | 0.76 | 0.387 |
| SP × ST | 1 | 60 | 33.32 | **<0.001** |
| Soil × ST | 1 | 60 | 0.00 | 0.981 |
| SP × C | 1 | 60 | 1.54 | 0.220 |
| Soil × C | 1 | 60 | 3.69 | 0.059 |
| ST × C | 1 | 60 | 0.08 | 0.777 |
| SP × Soil × ST | 1 | 60 | 1.70 | 0.197 |
| SP × Soil × C | 1 | 60 | 0.02 | 0.877 |
| SP × ST × C | 1 | 60 | 13.86 | **<0.001** |
| Soil × ST × C | 1 | 60 | 0.06 | 0.808 |
| SP × Soil × ST × C | 1 | 60 | 1.54 | 0.220 |
| (B) *Belowground biomass*^1^ | | |  |  |
| Species (SP) | 1 | 28 | 150.36 | **<0.001** |
| Soil type (Soil) | 1 | 28 | 2.87 | 0.102 |
| Sterilization (ST) | 1 | 28 | 197.92 | **<0.001** |
| SP × Soil | 1 | 28 | 0.09 | 0.763 |
| SP × ST | 1 | 28 | 4.19 | 0.050 |
| Soil × ST | 1 | 28 | 1.79 | 0.192 |
| SP × Soil × ST | 1 | 28 | 8.50 | **0.007** |

^1^ Data were based on the aboveground biomass and belowground biomass of *A. odoratum* and *C. jacea* on soils conditioned by monocultures of *A. odoratum* and *C. jacea* (monospecific soils) in the field.

**Table S2.** Results of mixed-effect ANOVA testing the effects of soil type (“own” vs. “foreign” soil), sterilization (live vs. sterile) and competition (monoculture vs. mixture; only for aboveground biomass) on aboveground biomass (A) and belowground biomass (B) of *A. odoratum* and *C. jacea* in the greenhouse experiment. DF, DenDF, *F-* and *P*-values are given.

|  |  |  | *A. odoratum* | |  | *C. jacea* | |
| --- | --- | --- | --- | --- | --- | --- | --- |
|  | DF | DenDF | *F* | *P* |  | *F* | *P* |
| (A) *Aboveground biomass*^1^ | | |  |  |  |  |  |
| Soil type (Soil) | 1 | 28 | 3.22 | 0.084 |  | 8.34 | **0.007** |
| Sterilization (ST) | 1 | 28 | 166.22 | **<0.001** |  | 11.18 | **0.002** |
| Competition (C) | 1 | 28 | 7.91 | **0.009** |  | 0.57 | 0.456 |
| Soil × ST | 1 | 28 | 1.12 | 0.298 |  | 0.86 | 0.362 |
| Soil × C | 1 | 28 | 2.74 | 0.109 |  | 1.63 | 0.212 |
| ST × C | 1 | 28 | 7.52 | **0.011** |  | 8.41 | **0.007** |
| Soil × ST × C | 1 | 28 | 1.40 | 0.246 |  | 0.52 | 0.477 |
| (B) *Belowground biomass*^1^ | | |  |  |  |  |  |
| Soil type (Soil) | 1 | 12 | 2.90 | 0.114 |  | 0.84 | 0.378 |
| Sterilization (ST) | 1 | 12 | 189.03 | **<0.001** |  | 62.92 | **<0.001** |
| Soil × ST | 1 | 12 | 13.16 | **0.004** |  | 1.09 | 0.318 |

^1^ Data were based on the aboveground and belowground biomass of *A. odoratum* and *C. jacea* on soils conditioned by monocultures of *A. odoratum* and *C. jacea* (monospecific soils) in the field. Values with *P* <0.05 are in bold.

**Table S3.** Full model results of ANOVA testing the effects of species (*A. odoratum* vs. *C. jacea*), sterilization (live vs. sterile) and competition (monoculture vs. mixture; only for aboveground PSF) on the aboveground feedback (A) and belowground feedback (B) of *A. odoratum* and *C. jacea* in the greenhouse experiment. DF, DenDF, *F*-, and *P*- values are given.

|  | DF | DenDF | *F* | *P* |
| --- | --- | --- | --- | --- |
| (A) *Aboveground PSF* | |  |  |  |
| Species (SP) | 1 | 28 | 0.94 | 0.342 |
| Sterilization (ST) | 1 | 28 | 0.00 | 0.979 |
| Competition (C) | 1 | 28 | 4.55 | **0.042** |
| SP × ST | 1 | 28 | 2.10 | 0.159 |
| SP × C | 1 | 28 | 0.03 | 0.864 |
| ST × C | 1 | 28 | 0.07 | 0.788 |
| SP × ST × C | 1 | 28 | 1.90 | 0.180 |
| (B) *Belowground PSF* | |  |  |  |
| Species (SP) | 1 | 12 | 0.11 | 0.750 |
| Sterilization (ST) | 1 | 12 | 2.05 | 0.178 |
| SP × ST | 1 | 12 | 9.75 | **0.009** |

**Fig. S1.** Replacement diagram of the aboveground biomass of *A. odoratum* and *C. jacea* in the field experiment. The initial total seedling density was 144 seedlings/plot. Data of aboveground biomass was based on the central 60 × 60 cm^2^ field collected after three growing seasons in 2015.

**Fig. S2.** Relationship between the relative crowding coefficient (*k*) of *A. odoratum* (A) and *C. jacea* (B) and their planting frequency in the mixtures in the field plot. *F*-, *R*^2^- and *P*-values based on linear regressions are given.

**Fig. S3.** Feedback strength (log-ratio of biomass in “own” and “foreign” soil) of *A. odoratum* and *C. jacea* in monocultures and mixtures. “Sterile” and “Live” indicate sterilized soil and non-sterilized soil respectively, respectively. Plants were grown in monocultures and in 1:1 mixtures in the greenhouse experiment. Mean values (± 1 SE) and significant effects of an ANOVA with sterilization (ST), competition (C; only for aboveground biomass) and the interaction are also presented: ^*^ *P*<0.05 (see Table S3 for full analysis).

**Fig. S4.** Relationship between aboveground biomass per plant of *A. odoratum* in monocultures (A and B) or in 1:1 mixtures (C and D) in the greenhouse experiment and its aboveground biomass in the field plots. Black and white dots represent soils collected from field plots planted with monocultures and mixtures, respectively. *F*-, *R*^2^- and *P*-values based on linear regressions are given.

**Fig. S5.** Relationship between aboveground biomass per plant of *C. jacea* in monocultures (A and B) or in 1:1 mixtures (C and D) in the greenhouse experiment and its aboveground biomass in the field plots. Black and white dots represent soils collected from field plots planted with monocultures and mixtures, respectively. *F*-, *R*^2^- and *P*-values based on linear regressions are given.

**Fig. S6.** Relationship between belowground biomass per soil core of *A. odoratum* (A and B) or *C. jacea* (C and D) in monocultures in the greenhouse experiment and its aboveground biomass in the field plots. Black and white dots represent soils collected from field plots planted with monocultures and mixtures, respectively. *F*-, *R*^2^- and *P*-values based on linear regressions are given.

**Fig. S7.** Relationship between the biomass of *A. odoratum* in the field plot and competitive balance (CB;$\ln\frac{{MIX}_{Ao}}{{MIX}_{Cj}}$) between *A. odoratum* and *C. jacea* in the 1:1 mixtures in live soil in the greenhouse experiment after removing of an influential data point. Negative values indicate biomass of *C. jacea* is higher while positive values indicate *A. odoratum* biomass is higher. Black and white dots represent soils collected from field plots planted with monocultures and mixtures, respectively. *F*-, *R*^2^- and *P*-values based on linear regressions are given.

**Fig. S8.** Relationship between the total biomass per pot of *A. odoratum* and *C. jacea* and competitive balance ($\ln\frac{{MIX}_{Ao}}{{MIX}_{Cj}}$) between *A. odoratum* and *C. jacea* in the 1:1 mixtures in greenhouse experiment. *F*-, *R*^2^- and *P*-values based on linear regressions are given.

**Methods S1** Description of soil chemical analysis.

The amount of NH_4_, NO_3_ and PO_4_ (mg/kg dry soil sample) were determined by adding 30.0 ml of 0.01 mol/L CaCl_2_ solution to soil samples (3.0 g), shaking mechanically for at least 2 h at room temperature (20 ℃), filtering the solution and analyzing the nutrients in the soil extracts in a Skalar Segmented Flow Analyzer. Soil pH-H_2_O was determined by adding 25.0 ml demi-water to soil samples (volume 5.0 ml), shaking for 5 min and wait 2 h before measuring. Soil organic matters were determined by measuring the difference between weights of the oven-dried (105 ℃) soil samples (5.0-10.0 g) before and after being heated in a furnace at 550 ℃. Weights of soil samples were determined and recorded after cool down in the air to handwarm and further cool for at least 45 min in a desiccator. Soil moisture content was determined by measuring the difference between the weights of soil samples before and after oven-dried (105 ℃) in the oven.
